# Environmental factors and gut microbiota: Toward better conservation of deer species

**DOI:** 10.3389/fmicb.2023.1136413

**Published:** 2023-03-07

**Authors:** Yu Wang, Bo Xu, Huan Chen, Fang Yang, Jinlin Huang, Xin’an Jiao, Yunzeng Zhang

**Affiliations:** ^1^Jiangsu Co-Innovation Center for Prevention and Control of Important Animal Infectious Diseases and Zoonoses, Yangzhou University, Yangzhou, China; ^2^Jiangsu Key Laboratory of Zoonosis, Yangzhou University, Yangzhou, China; ^3^Joint International Research Laboratory of Agriculture and Agri-Product Safety of the Ministry of Education, Yangzhou University, Yangzhou, China; ^4^Key Laboratory of Prevention and Control of Biological Hazard Factors (Animal Origin) for Agri-Food Safety and Quality, Ministry of Agriculture of China, Yangzhou University, Yangzhou, China

**Keywords:** deer species, gut microbiota, environmental factors, conservation, microbial composition

## Abstract

Thousands of microbial species inhabiting the animal gut, collectively known as the gut microbiota, play many specific roles related to host nutrient metabolism and absorption, immune regulation, and protection from pathogenic bacteria. Gut microbiota composition is affected by several internal and external factors, such as the host genotype, dietary intake, breeding environment, and antibiotic exposure. As deer species are important members for maintaining ecosystem balance, understanding the effects of multiple factors on the gut microbiota of deer species, particularly endangered ones, is crucial. In this review, we summarize and discuss the factors that significantly affect the gut microbiota of deer and present the impacts of these factors on microbial composition. In particular, we focused on the changes in gut microbiota due to dietary differences under different conditions, including seasonal changes, different geographical locations, and captivity, as well as weaning and pathogen disturbance. Understanding the correlations between gut microbiota composition and its driving factors is important for evaluating and improving the captive breeding environment for better conservation of endangered deer species, and reintroducing wild deer populations in the future.

## 1. Introduction

Several species affiliated with *Cervidae* and the primitive deer *Moschus* spp. (collectively referred to as deer species hereafter) are known to play a vital role in enriching the dense forest biodiversity and maintaining ecosystem balance (Li et al., 2016; Spake et al., 2020). However, recent climate changes and human activities have led to the endangerment or even extinction of many wild animals, including deer, from forest systems (Cai et al., 2020). At present, numerous deer species are listed as endangered, vulnerable, or highly endangered by the International Union for Conservation of Nature Red List of Threatened Species and as critically endangered by the Red List of China’s Vertebrates (Jiang et al., 2016); these include musk deer (*Moschus* [*M.*] spp.) (Harris, 2016), Père David’s deer (*Elaphurus* [*E.*] *davidianus*) (Zhang et al., 2017), sika deer (*Cervus* [*C.*] *nippon*) (Guan et al., 2017), and white-lipped deer (*C. albirostris*) (Harris, 2016). Both captive breeding and *ex situ* conservation have been effectively applied for maintaining and restoring these endangered deer species, with considerable success being achieved (Wang et al., 2016; Sun et al., 2020). However, increasing numbers of studies have reported subhealth conditions of many captive animals, including deer, and even mass die-offs in protected areas, mainly resulting from gastrointestinal infections (Li et al., 2022).

The gut microbiota, which consists of trillions of microorganisms (including archaea, bacteria, fungi, and viruses), plays crucial roles in the health, physiology, and development of the host and is thus recognized as an integral part of the animal holobiont (Hugon et al., 2017). The gut microbiota is not constant and differs among individuals; it is susceptible to various internal and external factors, such as the host genotype, dietary intake, lifestyle, breeding environment, and antibiotic exposure (Li Y. et al., 2017; Wang et al., 2022). Notably, certain members of the gut microbiota play more important roles than the remaining commensal ones, e.g., conferring resistance to pathogens and facilitating food digestion; moreover, several isolates with desired beneficial functions have been obtained through omics-guided microbiota analysis and targeted microbial isolation approaches (e.g., ref. Zipperer et al., 2016; Yuan et al., 2022). Beneficial microbe administration-based microbiota manipulation approaches mentioned below have consequently been developed and have shown promising benefits in animal breeding and raising practices (Anee et al., 2021). With such rapid developments in omics technologies and their contributions to microbiota decryption and application, several omics-based studies have been conducted for analyzing the gut microbiota of deer species, particularly six species belonging to the family *Cervidae* [sika deer (*C. nippon*) (Guan et al., 2017; Wang et al., 2022), Père David’s deer (*E. davidianus*) (Zhang et al., 2018; Sun et al., 2019), red deer (*C. elaphus*) (Menke et al., 2019; Wang et al., 2019), white-lipped deer (*C. albirostris*) (Li J. G. et al., 2017; Li et al., 2022; You et al., 2022), Siberian roe deer (*Capreolus pygargus*) (Liu J. et al., 2019), and white-tailed deer (*Odocoileus virginianus*)] (Delgado et al., 2017; Minich et al., 2021) and three species belonging to the genus *Moschus* [alpine musk deer (*M. chrysogaster*) (Sun et al., 2020), Siberian musk deer (*M. moschiferus*) (Su et al., 2022), and forest musk deer (*M. berezovskii*)] (Li Y. et al., 2017). The predominant bacterial phyla in these deer species were found to be *Firmicutes*, *Bacteroidetes*, *Proteobacteria*, and *Actinobacteria*; these findings are consistent with previous findings regarding the gut microbiota of ruminants (Tanca et al., 2017). Furthermore, these studies also demonstrated that the gut microbiota composition of deer was dramatically changed under the effects of environmental factors. However, comprehensive understanding of the effects of environmental factors on the gut microbiota of deer species is still lacking. In this minireview, we summarize and discuss recent findings regarding the gut microbiota of deer species using omics approaches, mainly focusing on how the gut microbiota structure is affected by captivity-, season-, and geographical location-related dietary changes; weaning; and the presence of pathogens. Our findings can benefit the development of microbiota optimization-based approaches to improve the captive breeding and raising processes of endangered deer populations.

## 2. Effects of diets on the gut microbiota of deer species

Among the factors that are known to dramatically affect the gut microbiota, particular attention has been paid to diet and its role in shaping the composition and function of the gut microbiota (Bibbò et al., 2016). Because of changes in dietary nutrients (e.g., fiber, starch, proteins, and fats), the taxa that prefer the given nutrients usually exhibit higher growth and proliferation rates, resulting in rapid alteration of the gut microbiota composition. However, a large fraction of microbes can still be remarkably stable in healthy individuals for years (Fassarella et al., 2021). In recent years, several studies have been conducted to understand the effects of diet changes on the gut microbiota of deer species, mainly by comparing the gut microbiota compositions of individuals in different seasons and geographical locations and those of captive and wild individuals, and are discussed below in detail.

### 2.1. Diet changes due to seasonal changes

**Figure 1:**
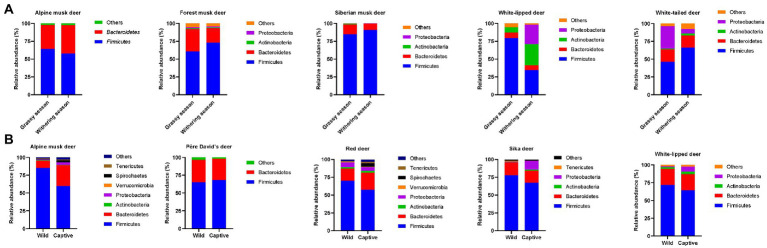
Microbial composition of the gut bacterial community of deer species at the phylum level in distinct seasons **(A)**, and wild and captive environments **(B)**, respectively.

Food resources can change over temporal scales. For the deer population, sufficient and diverse fresh plant-derived food is available in the grassy season; however, food resources and choices are relatively limited in the withering season due to severe weather conditions (Hu et al., 2018; Li et al., 2022; Su et al., 2022; You et al., 2022). Dramatic seasonal variations in gut microbiota compositions are observed in deer species (Figure 1A). The relative abundance of *Bacteroidetes* is significantly higher in the grassy season than in the withering season. Members affiliated with *Bacteroidetes* are known to play a key role in degrading high-molecular-weight organic materials, including carbohydrates and proteins (Jami et al., 2013), thereby improving the nutritional composition of the host. This is consistent with the fact that the protein, starch, and lactate contents are higher in fresh leaves available in the grassy season than in limited foods available in the withering season (Hu et al., 2018). In contrast, the relative abundance of *Firmicutes* and the *Firmicutes*/*Bacteroidetes* (F/B) ratio are higher in the withering season than in the grassy season. *Firmicutes* can digest and absorb nutrients by degrading diverse substances, and the gut microbiota with a high F/B ratio can exhibit a higher fermentation efficiency and thus obtain more energy from food (Chevalier et al., 2015; Su et al., 2022). Moreover, a high F/B ratio can promote fat deposition in the host (Su et al., 2022), which is important for adapting to the cold withering season. However, these recent studies were conducted using 16S rDNA amplicon-based analyses, which hampered the identification of the key microbes and associated functions that are responsible for plant-derived substance degradation and energy conversion, and whole-genome-based metagenomics, metagenome-assembled-genome recovery and analysis, and culturomics can be performed to bridge these knowledge gaps (Stewart et al., 2019; Thomas et al., 2021; Whon et al., 2021).

### 2.2. Diet changes due to geographical locations

The climatic conditions, including temperature, precipitation, and vegetation, usually vary dramatically among different geographical locations. The gut microbiota compositions of forest musk deer species from Sichuan (subtropical monsoon climate) and Qinghai (highland continental climate, higher latitude, and lower temperature than Sichuan) differ significantly (Liu X. et al., 2019). Moreover, the Père David’s deer populations living in Shishou (subtropical monsoon climate) and Beijing (semihumid monsoon climate, higher latitude, and lower temperature than Shishou) harbor very different gut microbiota, with the gut microbiota of deer in Beijing exhibiting a higher F/B ratio than that of deer in Shishou (Zhang et al., 2018). These observed gut microbiota differences are probably associated with the available vegetation and temperature variations among geographic locations, the high abundance of *Firmicutes* and higher F/B ratio in the gut microbiota of deer species living in the geographic location with lower temperature may benefit the host given that the gut microbiota with a high F/B ratio usually exhibit a higher fermentation efficiency and thus the host can obtain more energy from food to maintain the body temperature (Chevalier et al., 2015; Su et al., 2022). Again, species- and strain-level resolution-based microbiota analysis can be performed to identify the key microbial members and their key functional traits involved in geographic location-associated diet and temperature adaptation of deer species.

### 2.3. Diet changes due to captivity

Captive breeding and raising have been implemented for several endangered deer species. The formulated forage provided to captive deer species is usually dramatically different from the food available in the wild (Li Y. et al., 2017). Consequently, the gut microbiota structure, particularly the F/B ratio, is very different between captive and wild deer populations (Guan et al., 2017; Sun et al., 2019, 2020, 2021; Jiang et al., 2021; Li et al., 2022). The relative abundance of *Firmicutes* is significantly higher in wild deer species than in captive ones, while the relative abundance of *Bacteroidetes* exhibits an opposite trend (Figure 1B). Therefore, the F/B ratio is higher in wild deer species than in captive ones. This difference in F/B ratio in the gut microbiota between wild and captive deer populations probably reflects the fact that a diverse diet spectrum, mostly consisting of various high-fiber leaves, is accessible to wild deer species, while a diet predominately consisting of fresh leaves supplemented with formulated foods containing high carbohydrate and protein concentrations is available to captive deer species. Notably, a higher relative abundance of *Proteobacteria* is found in captive sika deer and white-lipped deer than in wild ones (Li et al., 2022; Wang et al., 2022). Gut microbes belonging to *Proteobacteria* are known to degrade lignin and other various ingredients (Fang et al., 2012), further suggesting the effects of an artificially formulated diet on the gut microbiota structure of captive deer species. However, an increase in *Proteobacteria* in captive deer species could also indicate an increased risk of intestinal disorders because many *Proteobacteria*-affiliated gut bacteria are known pathogens or potential pathogens (Joat et al., 2021). Although the gut microbiota has certain plasticity that can help the host adapt to changes from natural to captive dietary supplies, some potential health risks, such as a decrease in nutrient absorption efficiency and an increase in potentially pathogenic bacteria, among captive populations cannot be ignored (Gogarten et al., 2012). Therefore, monitoring the digestive system of captive deer species and understanding whether the deer species have adapted to artificial diets and new environments are important for wildlife conservation.

## 3. Effects of weaning on the gut microbiota of deer species

**Figure 2:**
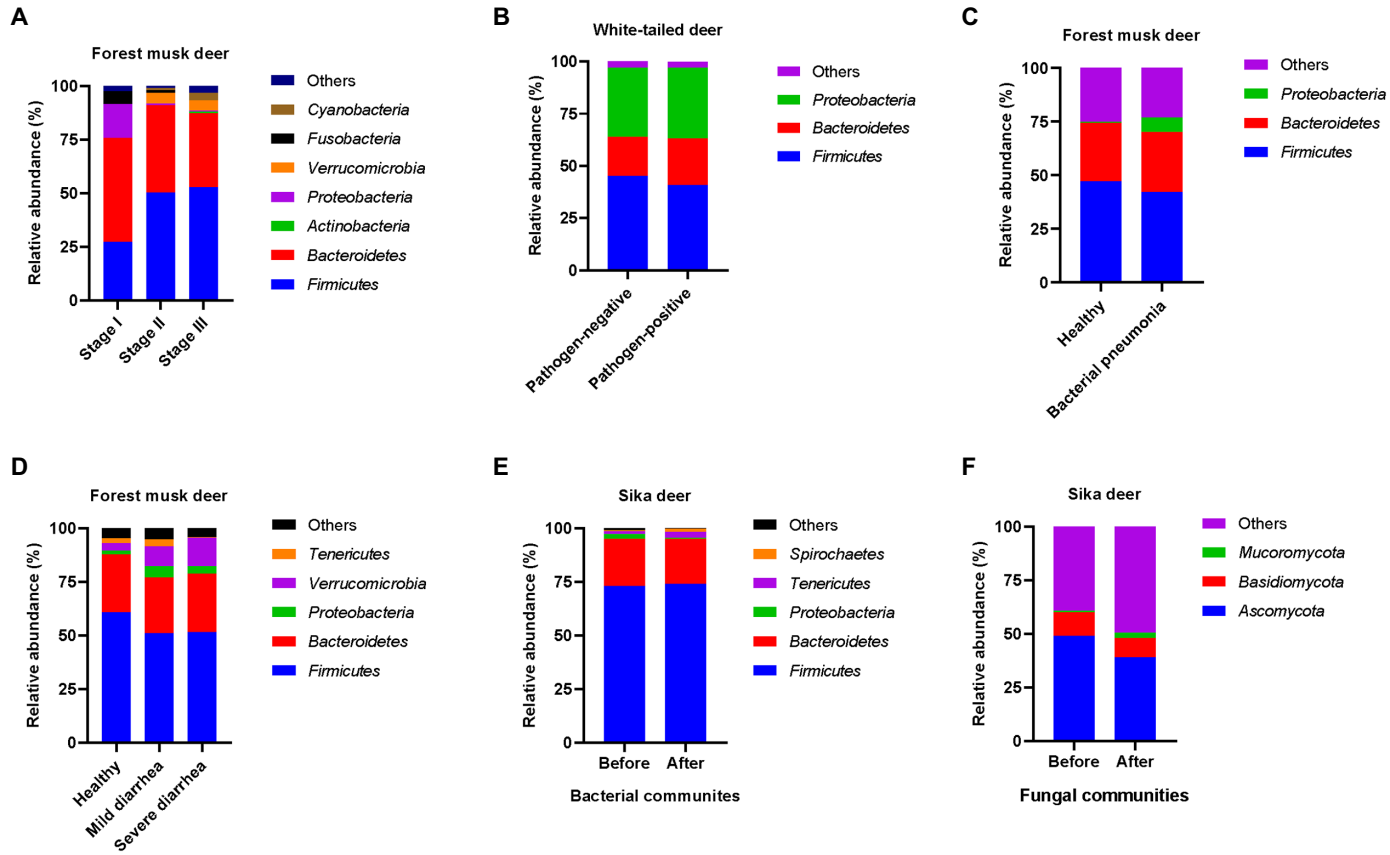
Microbial composition of the gut bacterial community of deer species at the phylum level in weaning **(A)**, pathogen-positive or -negative **(B-D)**, and antibiotic treatments **(E,F)**, respectively.

Weaning, a process of replacing milk feeding with an increasing range of ingested nutrients, is an important event in the early life of mammals (Li et al., 2020). It is usually accompanied by dramatic changes in the composition of the gut microbiota. Special interest has been paid to the gut microbiota dynamics of humans and several animals during the weaning period. Recent studies on forest musk deer revealed that *Proteobacteria* maintained a relatively high abundance when infants were solely fed milk (stage I), while its relative abundance decreased with an increase in the relative abundance of *Firmicutes* in the gut microbiota from stage I to stage II (when milk feeding reduced and plant leaves and feed concentrate were added) and stage III (when only leaves and feed concentrate were fed; Figure 2A; Li et al., 2020, 2021). *Proteobacteria* is ubiquitous and abundant in the intestines of breastfed infants, whereas *Firmicutes* are capable of digesting and absorbing nutrients from diverse substrates, including fiber-rich plant leaves. Notably, the gut microbiota composition does not differ significantly between stage II and stage III in weaning deer species, suggesting that the establishment of the gut microbiota is preliminarily completed before total weaning. Similar gut microbiota succession patterns have been observed in human infants (Bäckhed et al., 2015), pigs (Kim et al., 2012), and horses (Mach et al., 2017). Hence, careful attention should be paid to the formula of plant leaves and feed concentrate fed to deer species during the weaning process in order to enable a more healthy and mature gut microbial composition. However, the microbial members and functions important for gut microbiota reassembly and maturity during the weaning process need to be explored further.

## 4. Effects of pathogens and antibiotic treatments on the gut microbiota of deer species

The presence of pathogens in the gut microbiota usually alters the microbiota composition, even if the host is asymptomatic. White-tailed deer containing diarrheagenic *Escherichia coli* in their gut microbiota harbored an altered microbiota structure compared with those not containing *E. coli* (Delgado et al., 2017; Figure 2B). Moreover, Li et al. (2018) compared the gut microbiota of healthy forest musk deer and those with mild and severe diarrhea, and observed that their gut microbiota compositions differed, with a lower relative abundance of *Firmicutes* and higher relative abundance of *Proteobacteria* being noted in the gut microbiota of diseased individuals than in that of healthy ones (Li et al., 2018; Figure 2D). Their study also identified *Escherichia*/*Shigella* and *Fusobacterium* as the potential causal agents of diarrhea, suggesting that metagenomics-based microbiota profiling can be a powerful tool to identify the causal agents of infective diseases. Interestingly, forest musk deer with and without bacterial pneumonia were found to harbor different gut microbiota compositions (Zhao et al., 2021; Figure 2C), although the pathogen that causes pneumonia does not directly interact with the gut microbiota. The roles of gut microbes that exhibit positive and negative correlations with pneumonia can be further explored in order to develop gut microbiota manipulation approaches for preventing and/or treating pneumonia. Antibiotic administration is a widely used treatment for controlling bacterial infections. The relative abundance of gastrointestinal pathogenic bacteria together with that of Proteobacteria phyla in the gut microbiota of sike deer dramatically decreased after antibiotic treatment (Hu et al., 2020; Figure 2E). However, the fungal content (Hu et al., 2020; Figure 2F) in the gut microbiota significantly changed from that before antibiotic treatment. The effects of such changes in fungal community compositions in the gut microbiota on the hosts need to be studied further.

## 5. Conclusion and future outlook

The health of an animal is inevitably associated with the stability of its gut microbiota. Throughout the lifespan of deer, their gut microbiota composition is affected by various factors, such as diet, living environment, antibiotic use, and diseases. A thorough understanding of how the gut microbiota is affected by the given factors through high-throughput sequencing can enable a more reliable assessment of the effects of various factors on gut microbiota composition and on host development and health. The relative abundance of *Firmicutes* and *Bacteroides* and the F/B ratio in the gut microbiota are important for deer species to adapt to their habitats and are mainly determined by amplicon sequencing. Studies identifying the microbial members and metabolic functions that play key roles in host adaptation, including digestion of the given feed, low temperature adaptation, and health maintenance, are urgently needed. A few whole-genome metagenomic studies have assessed the gut microbiota of deer and identified several functional genes and genome sequences of beneficial microbes (Su et al., 2022; Wang et al., 2022). With the rapid development of high-throughput sequencing, bioinformatics, culturomics, and *in situ* microbiota editing technologies, the gut microbiota can be manipulated to help the host adapt to the environment (e.g., efficiently digest food and exhibit antagonistic effects against pathogens; Stewart et al., 2019; Thomas et al., 2021; Whon et al., 2021). The development of gut microbiota research will contribute to the conservation of deer species, particularly endangered ones, and benefit future wild population restoration programs.

## Author contributions

YZ and YW conceived and revised the manuscript. BX, HC, and FY contributed reference download and its organization. JH, XJ, and YZ contributed funding acquisition. All authors have read and agreed to the published version of the manuscript.

## Funding

This work was funded by the Yangzhou University Interdisciplinary Research Foundation for Veterinary Medicine Discipline of Targeted Support (grant number yzuxk202003), the Postgraduate Research and Practice Innovation Program of Jiangsu Province (grant number KYCX21_3208), the Priority Academic Program Development of Jiangsu Higher Education Institutions (PAPD) and 111 Project (D18007).

## Conflict of interest

The authors declare that the research was conducted in the absence of any commercial or financial relationships that could be construed as a potential conflict of interest.

## Publisher’s note

All claims expressed in this article are solely those of the authors and do not necessarily represent those of their affiliated organizations, or those of the publisher, the editors and the reviewers. Any product that may be evaluated in this article, or claim that may be made by its manufacturer, is not guaranteed or endorsed by the publisher.
